# Self-We-Others Schemata Differentiation as a Base for Personal Agency and Social Attitudes

**DOI:** 10.3389/fpsyg.2016.01227

**Published:** 2016-08-18

**Authors:** Maria Jarymowicz, Anna Szuster

**Affiliations:** Faculty of Psychology, University of WarsawWarsaw, Poland

**Keywords:** self-distinctness, self-control, implicit priming, resistance to implicit affect, in-group vs. out-group attitudes

The extent to which the Self-schema is differentiated from the cognitive schemata representing other people has important meaning for diversification of social vs. individual identity (Snyder and Fromkin, 1980; Brewer, 1991; Jarymowicz, 1993), socialization and individuation (Ziller, 1964), and interdependence and individualism (Waterman, 1981). All these concepts must be taken into account in order to gain a better understanding of the social world (Greenwald and Pratkanis, 1984; Baumeister, 2005; Leary and Tangney, 2013).

Cognitive individuation is connected with a subject's individuality. Unfortunately, this concept is sometimes treated as being synonymous with an individualistic orientation, in the sense of the possession of egocentric goals. In the Chinese language, the notion of individuality is replaced by selfishness (Nisbett, 2003). Yet it is evident that all human decisions and actions involve the ego to some degree and that ego-involvement is imperative for the realization of goals (Allport, 1955)—all goals, whether they are personal or social. The concept of individuality can be used to refer to self-awareness and a clear view of personal preferences and goals, as well as to the ability to delay gratification and to act in a socially responsible way.

## The main assumptions

The aim of this paper is to contribute some empirical evidence to the analysis of the relationship between the Self-schema and social functioning. Our studies were based on the following assumptions (Jarymowicz, 1987; Jarymowicz and Szuster, 2014): (1) individuality results from the individuation process, which involves differentiation between the Self-Other schemata and leads to lower or higher Self-schema distinctness; (2) Self-distinctness is a prerequisite of self-awareness and personal agency, as well as of decentration and the capacity to understand other people (Piaget, 1965, 1973); (3) Self-distinctness—based on Self vs. We schemata differentiation—allows transgression of the in-group perspective and reduction of in-group favoritism.

## Self as the subject vs. self as the distinct object of knowledge

The Self duality is considered crucial to personal agency (Reykowski, 1979; Epstein, 1980; Greenwald and Pratkanis, 1984; Jarymowicz, 2008; Leary and Tangney, 2013). An individual becomes the object of self-cognition, self-esteem, and self-control, capable of recognizing the world from a non-egocentric perspective (Markus, 1977; Bandura, 1982; Reykowski, 1989; Kozielecki, 1997; Sedikides and Skowronski, 2003; Baumeister, 2005; Gazzaniga, 2011). The studies discussed herein focus on the relationships between the degree of the Self-distinctness, the efficacy of self-control, and the various attitudes that are displayed toward out-group members.

We drew from research on social comparison processes in the operationalization of the Self-schema distinctness. Numerous data have implicated that estimating the degree of Self-Other similarity depends on the motivation to be similar/dissimilar to/from others (Suls and Miller, 1977). Some results showed that whilst the Self-Other similarity is attractive, so too is the difference. It was found that an unconscious tendency exists to seek out diversity and uniqueness (Fromkin, 1972; Codol, 1979; Snyder and Fromkin, 1980), and that the degree of similarity is often underestimated (Codol and Jarymowicz, 1984). In our research, we distinguished between: (1) assessment of the Self-Other similarity by an individual; and (2) similarity between the attributes ascribed by an individual to the Self and to Others during the course of a procedure that did not involve explicit comparisons.

## Measurement of self-distinctness

The Questionnaire of Social Perception (Jarymowicz, 1993) was developed to measure the Self-schema distinctness. This technique consists of three parts, which are successively handed to the participants in order to focus their attention on: (1) Others; (2) the We category; and (3) the Self. The questionnaire contains a list of 70 human traits (like creativity, honesty, rationality). The list is used three times.

1. Each participant first chooses the 10 traits to which she/he refers (“most often”) when thinking about other people.
2. In the next stage, the participant first has to indicate who the people are that she/he refers to as “we”, and is then required to select 10 traits that she/he recognizes in reference to “we.”
3. Finally, the participant selects the 10 traits referred (“most often”) to the Self.

Two indices of Self-distinctness are computed: (1) the number of unrepeated traits ascribed to the Self vs. to Others (SOD); and (2) the number of unrepeated traits ascribed to the Self vs. to the We (SWD). The latter SWD indices were used to test the hypothesis predicting that Self-We cognitive distinctness is necessary to limit the tendency to in-group favoritism.

## Research on the relationship between self-distinctness and personal agency

Wolak (1993) showed that the efficacy of emotional control training is related to Self-distinctness. His hypotheses were based on reports stating that Self-identity correlates with the efficacy of self-control (e.g., Organ, 1973). In Wolak's laboratory studies, unexpected sounds were applied to provoke emotional tension and the participants were requested to try to relax as quickly as possible. Changes in the number of spontaneous fluctuations and skin resistance were measured throughout. The emotional control training consisted of giving the participants some feedback on how effective they were at reducing level of tension. After the accurate feedback-based training, most of the participants were better at controlling their tension. However, when false feedback was provided in the next stage of the experiment, only the participants with high SOD indices displayed the same level of self-control improvement as in the previous stage.

We assumed that the Self-schema development leads to a stage wherein self-control becomes effortless and automatic. Some implications were tested by use of the implicit affective priming paradigm (Murphy and Zajonc, 1993; Ohme, 2007), which involves subliminal exposure to affective stimuli (faces displaying expressions of disgust or joy). Such implicit priming usually influences the subsequent evaluation of explicitly exposed neutral stimuli (unfamiliar Chinese ideograms): neutral stimuli are rated positively or negatively, depending on the valence of the implicitly primed stimulus (Kobylińska and Karwowska, 2014). In some of our studies (Jarymowicz, 2008), we used a modification of the affective priming paradigm to measure the implicit self-reference effect (Błaszczak and Imbir, 2012). There, the participants were presented with Chinese ideograms and told that they denoted human traits. They were then asked to estimate the degree to which a given ideogram described them. As a result, the participants were more likely to ascribe positively primed ideograms to themselves than negatively primed ones. But we also found that the magnitude of this effect varied with the level of Self-distinctness. The higher the SOD index of a subject, the lower was the implicit self-reference effect, thus, the less likely she/he was to attribute neutral, unfamiliar ideograms to her or himself. In other words, the data showed that some people are resistant to affective priming and fail to relate irrelevant stimuli to themselves (even those exposed implicitly).

To explore this finding, we turned to literature on implicit information processing (Uleman and Bargh, 1989; Reber, 1993; Underwood, 1996; Holyoak and Morrison, 2005). We hypothesized that some people have some insight into implicit information. To test this hypothesis, we used the implicit semantic priming paradigm: the subliminal exposition of words (Dobrenko and Jarymowicz, 2011). The participants were told that some words were invisible, and then subsequently asked to indicate which of the set of two explicitly exposed words—a synonym or a new word—corresponded to the word presented subliminally. A series of experiments showed that some participants were capable of recognizing the meaning of subliminally presented words, wherein they were more susceptible to choose the synonyms of the subliminal stimulus than unrelated words. In further studies, we demonstrated that he level of word recognition positively correlates with the Self-distinctive schema complexity (Jarymowicz et al., 2013).

## Research on the relationship between self-distinctness and social attitudes

Self-distinctness and the concept of individuality are often associated with diverse manifestations of egocentrism. For instance, twentieth century intercultural psychology characterized Eastern cultures as representing a conjunction of “interdependent” Self-schema and prosocial attitudes, whereas Western cultures were held to represent a combination of “independent” Self-schema, egocentric perceptions, and more or less egoistic behavior (Markus and Kitayama, 1991). Some authors try to argue that “prosocial orientation” often benefits the in-group (e.g., Triandis, 1989); we try to argue that attitudes toward in-group vs. out-group members depend on the Self-We-Others schemata differentiation (Jarymowicz, 1999).

In a study by Krzemionka (1993), participants were asked to declare their preferences with respect to 50 different activities and then shown the preferences of an unknown partner. In one condition, the “partner” was highly dissimilar; in the second condition, the “partner” was highly similar to the participant. Next, the participants estimated the attractiveness of this “partner”. The degree of Self-distinctness was also measured. Krzemionka showed that the estimations of partner attractiveness were related to SOD indices; more specifically, those participants with a low SOD rated similar partners as being more attractive than dissimilar partners, whereas no such differences were observed among participants with high SOD indices.

In studies taking into account the in-group vs. out-group opposition, we also found some confirmation that Self-distinctness plays a role in social perception (Jarymowicz, 2002, 2006). Some studies were based on the assumption that the similarity perceived between the Self and the Other corresponds in some way to the psychological self-other distance (Codol, 1979)—we found, for instance, that Polish participants underestimated their similarity to another person more frequently when the “other person” presented to them was German or Russian rather than a fellow countryman—but the effect was not significant in groups of participants with the highest relative level of Self-distinctness (of the SWD indices).

In numerous other studies, we found that effects related to in-group vs. out-group divisions correlate clearly with the Self-We differentiation. In one study (Jarymowicz, 2006), the participants were asked to read the positive opinions (condition 1) or the negative opinions (condition 2) of foreigners about Poles. They were then requested to generate (“as much as possible”) the positive and negative traits of Poles and Russians. In the first condition, the participants listed a similar number of positive and negative traits for both Poles and Russians. In the second condition, however, they showed a greater positive inclination toward Poles. Once again, this effect was only observed in participants with a relatively low SWD index.

A long and systematic series of studies on conspiracy beliefs carried out by Grzesiak-Feldman (2006) brought clear and coherent results. Data showed that Self-distinctness (the SWD indices) negatively correlated with conspiracy theory beliefs concerning the diverse activities of Jews.

Analogous effects were found in reference to individual representatives of in-group vs. out-group members. In an experimental study (Jarymowicz and Szuster, 2014), the participants were presented with a photograph of an attractive young woman and a brief description of her life and interests. In one condition, she was described as having participated in the Miss Polonia beauty pageant some years ago, while in the other condition, there was reference to her participation in the Miss Israel beauty pageant. In both conditions, the participants were asked several questions about the woman's skills and abilities. The main finding was that she was rated as being more competent when identified as being Polish rather than Israeli, but this effect of discrimination was only observed in those participants with a low SWD index.

## To summarize

Taken together, the results of the studies mentioned above indicate a certain degree of interdependence between the Self-schema distinctness and some aspects of personal agency (e.g., self-control and unconscious resistance to the influence of subliminal, irrelevant affective stimuli). Moreover, the results of the studies suggest that Self-distinctness (especially distinctness between the Self-We schemata) is an important determinant of the ability to perceive the Other from a non-egocentric or non-ethnocentric perspective. On the basis of the empirical data, we posit that Self-distinctness is a necessary (although not sufficient) condition of exocentric altruism and engaging for the benefit of other people—even those who are members of the out-group (Karyłowski, 1982; Szuster, 1994, 2005; Rutkowska and Szuster, 2011; Jarymowicz and Szuster, 2014).

## Author contributions

Contributions to the conception and design of the empirical works: MJ 70%, AS 30%. Drafting the work and revising it critically for important intellectual content: MJ 60%, AS 40%. Final approval of the version to be published: MJ 50%, AS 50%.

### Conflict of interest statement

The authors declare that the research was conducted in the absence of any commercial or financial relationships that could be construed as a potential conflict of interest.
